# ACSAuto-semi-automatic assessment of human vastus lateralis and rectus femoris cross-sectional area in ultrasound images

**DOI:** 10.1038/s41598-021-92387-6

**Published:** 2021-06-22

**Authors:** Paul Ritsche, Philipp Wirth, Martino V. Franchi, Oliver Faude

**Affiliations:** 1 Department of Sport, Exercise and Health, University of Basel, Basel, Switzerland; 2 Institute of Physiology, Department of Biomedical Sciences, University of Padua, Padua, Italy

**Keywords:** Muscle, Image processing

## Abstract

Open-access scripts to perform muscle anatomical cross-sectional area (ACSA) evaluation in ultrasound images are currently unavailable. This study presents a novel semi-automatic ImageJ script (named “ACSAuto”) for quantifying the ACSA of lower limb muscles. We compared manual ACSA measurements from 180 ultrasound scans of vastus lateralis (VL) and rectus femoris (RF) muscles to measurements assessed by the ACSAuto script. We investigated inter- and intra-investigator reliability of the script. Consecutive-pairwise intra-class correlations (ICC) and standard error of measurement (SEM) with 95% compatibility interval were calculated. Bland–Altman analyses were employed to test the agreement between measurements. Comparing manual and ACSAuto measurements, ICCs and SEMs ranged from 0.96 to 0.999 and 0.12 to 0.96 cm^2^ (1.2–5.9%) and mean bias was smaller than 0.5 cm^2^ (4.3%). Inter-investigator comparison revealed ICCs, SEMs and mean bias ranging from 0.85 to 0.999, 0.07 to 1.16 cm^2^ (0.9–7.6%) and − 0.16 to 0.66 cm^2^ (− 0.6 to 3.2%). Intra-investigator comparison revealed ICCs, SEMs and mean bias between 0.883–0.998, 0.07–0.93 cm^2^ (1.1–7.6%) and − 0.80 to 0.15 cm^2^ (− 3.4 to 1.8%). Image quality needs to be high for efficient and accurate ACSAuto analyses. Taken together, the ACSAuto script represents a reliable tool to measure RF and VL ACSA, is comparable to manual analysis and can reduce time needed to evaluate ultrasound images.

## Introduction

The morphology and architecture of a muscle is fundamental for its function^1^. In recent years, several studies highlighted the relation between architectural and morphological parameters of thigh muscles and performance^2–4^. For example, Evangelidis et al.^3^ demonstrated that muscle size is related to concentric, isometric and eccentric peak torque of the knee flexors and extensors.

Ultrasound is a relatively cheap and non-invasive tool to assess the architecture of muscles^5^ and its validity and reliability in laboratory settings are extensively documented^6, 7^. Among the morphological characteristics of a muscle, measurements of size through muscle volume, muscle thickness and anatomical cross-sectional area (ACSA) are most commonly used^8^. However, due to the size and shape of the quadriceps femoris muscles, conventional static B-mode ultrasound might be unsuitable to assess the ACSA of these muscles. This is mostly related to the limited field of view of the employed transducers^9, 10^. In this case, extended field of view (EFOV) ultrasound represents a valid solution^5, 6, 9^.

Ultrasound images are complex to evaluate and require a lot of effort and time for correct interpretation. Over the past few years, various automated and semi-automated programs were developed to support and accelerate the evaluation process of ultrasound images. These programs were mainly designed for image sequences derived from dynamic ultrasound measurements^11–13^. More recently, Seynnes and Cronin^14^ and Cronin et al.^15, 16^ published fully automated open source programs able to calculate fascicle length, pennation angle and thickness of muscles from static ultrasound pictures. Nonetheless, very few automated programs measuring the ACSA of a whole muscle in ultrasound images are currently available^15, 17^. For example, Salvi and colleagues^17^ used an image segmentation approach to measure ASCA of several muscles in static B-mode images. Chen et al.^15^ used a deep learning approach to train a neural network in evaluating m. rectus femoris (RF) ACSA in an EFOV image sequence acquired during voluntary contraction. Yet, none of these fully automated programs are openly accessible. In these regards, a reliable, openly accessible, semi-automatic program could decrease the effort and time needed to evaluate ACSA in ultrasound images. Additionally, the amount of subjective interpretation and image processing could be reduced and the comparability across studies increased.

In this study, we present the ACSAuto script, a semi-automatic script to measure ACSA in quadriceps muscles from ultrasound images. For the present study, the muscles of interest were RF and m. vastus lateralis (VL). From the quadriceps muscle, RF and VL are the most studied muscles in the literature^3, 6, 18–21^ and they represent more than half of the whole quadriceps volume^22^. Additionally, RF and VL ultrasound images usually demonstrate better quality compared to other quadriceps muscles. This facilitates the possibility of automatized measurements. The program is open source and runs in a single macro in FIJI^23^. FIJI is a distribution of ImageJ^24^ and is commonly used for ultrasound image analysis. To our knowledge, this represents the first open source attempt for the creation of a semi-automatic tool to assess ACSA of muscles from ultrasound images. We aimed (1) to describe the novel, semi-automatic ACSAuto script to measure ASCA in quadriceps muscles, to (2) compare it to a common manual image analysis approach and (3) to assess the inter- and intra-investigator reliability of the program.

## Methods

### Program description

**Figure 1:**
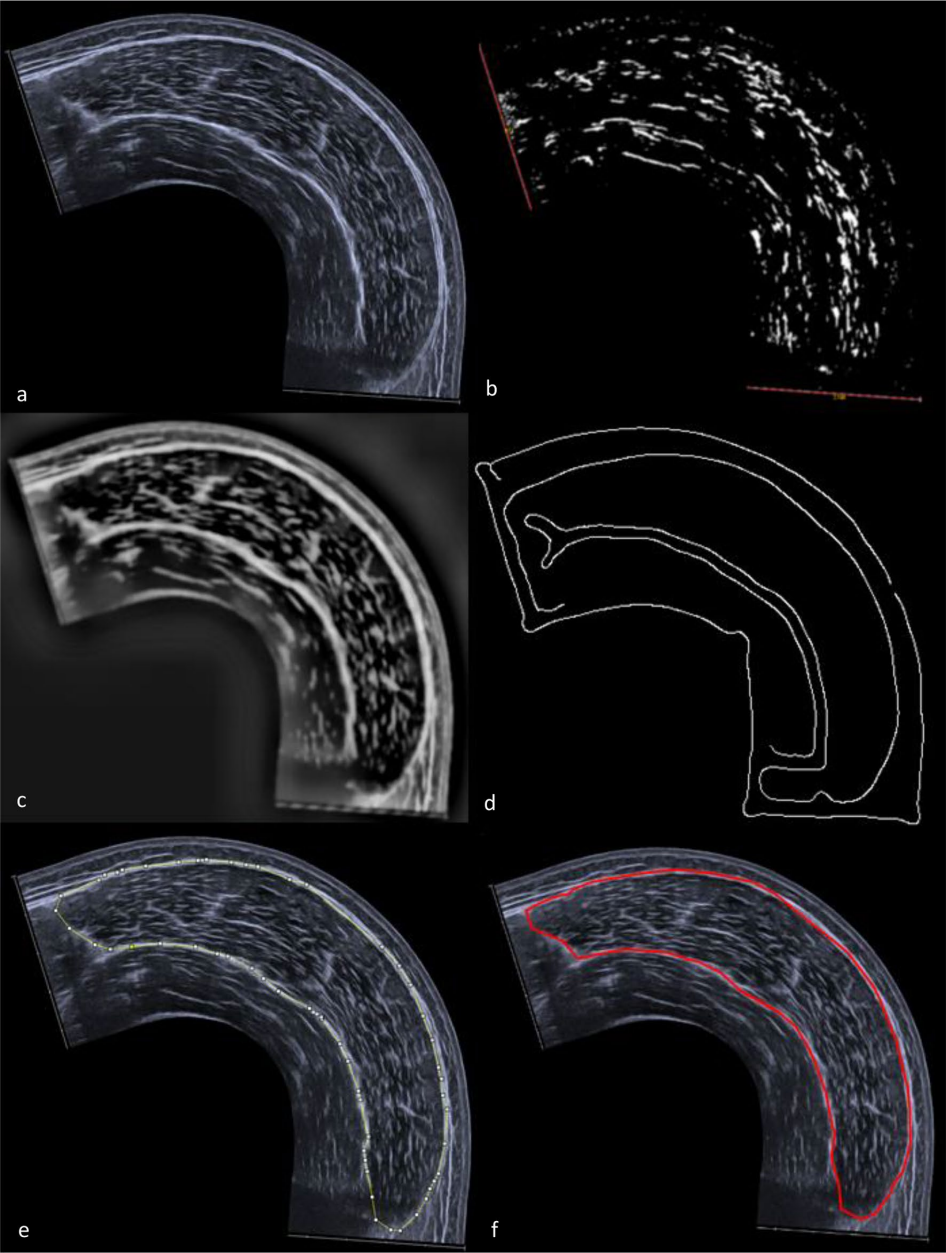
Workflow of the ACSAuto script: (**a**) raw Image, (**b**) automatic scaling using *ridge detection* plugin with red objects representing detected lines, (**c**) pre-processed Image, (**d**) mask for outline detection, placement of outline-finder starting points and removal of artefacts, (**e**) suggested muscle outlines, (**f**) measured area.

The ACSAuto script is based on the openly accessible Simple Muscle Architecture Analysis (SMA) macro developed by Seynnes and Cronin^14^. Our script consists of a single macro written in ImageJ 1.x macro language and runs in FIJI^23^. Two additional plugins (*Canny Edge Detector*^25^ and *Ridge detection*^26^) are needed to successfully run the script. The plugins can be installed the same way as the ACSAuto script (see supplementary material 2 ‘Installation guide’). Once the script is launched, a user interface opens where all relevant analysis parameters, such as image depth or muscle type, can be adjusted. When hovering the curser over a parameter, a short description is displayed. Either single images or whole folders containing image files can be processed. If a whole folder is processed (batch mode), input and output directories must be specified. So far, images can be processed in five different modalities. The “rectus femoris” and “vastus lateralis” modalities are designed for EFOV pictures containing only the respective muscle. The modalities “quad RF” and “quad VL” are designed for EFOV pictures containing both, RF and VL muscles, but only one muscle is measured. The modality “Quadriceps” measures the RF and VL ACSA in EFOV pictured containing both muscles. The script contains three different options to specify the outline-finder starting points (see section “Technical details”). Choosing the “manual” option, the user manually specifies one (RF) or three (VL) outline-finder starting points within the image. The “Fixed Pixels” option specifies the outline-finder starting points using hardcoded coordinates. Outline-finder starting points are estimated based on image width and height if the option “Automatic” is used. Image scaling is possible in two ways, but mandatory. If “automatic” is selected, the picture will be scaled automatically. If “manual” is selected, a line equally to the scanning depth needs to be drawn into the picture. The scanning depth must be specified. The analysis script is particularly designed for ultrasound pictures displaying the medial muscle border on the left and the lateral border on the bottom middle or right (Fig. 1a). If this is not the case, flipping options should be used. Otherwise “Fixed Pixels” and “Automatic” outline-finder strategies as well as the outline-finding process might fail. After all analysis parameters are specified, a dialogue window will appear asking for pre-processing settings (see section “Technical details”). Default values are modality- and muscle-dependent and are based on our sample pictures. During the analysis, three other dialogue windows appear. First, the user is asked to select and delete artefacts in the image. Secondly, if “Manual” outline-finder starting points was selected, the points must now be placed within the muscle. Last, the user is asked to adjust the region of interest. Manual adjustment of the selected muscle outline can be performed. The analysis results are displayed and accessible once an image is processed.

### Instructional material

An instructional video for the program can be found here: https://www.youtube.com/watch?v=b4D8TJKU-dI.

For a detailed written instruction on how to use the program see supplementary material 3.

### Technical details

The active image needs to be automatically or manually scaled. For automatic scaling, the active image is duplicated, thresholded, a mask created by automatic particle counting is subtracted and convolution filtering is applied. Then the *ridge-detection*^26^ plug-in searches for elongated objects of specific length within the image, for example a scaling line (Fig. 1b). Automatic scaling is only possible if some sort of scaling line is present in the image. The detected length range of automatic scaling is hardcoded based on our sample images.

In the first phase of the analysis process, the image is pre-processed using the earlier specified parameters (Fig. 1c). “*Min Length Fac*” describes a cut-off value relative to image width. If the length of an object is lower than this value, the object is removed from the image during pre-processing. The value of “*Tubeness sigma*” is used in the *Tubeness*^27^ plugin and either less or more “tube-like” objects in the image are detected and enhanced. This is used to detect the muscle aponeuroses^14^ (Fig. 1d). The *“Gaussian sigma*” value is used for smoothing of the image and applied in a convolution filter during pre-processing. The pre-processing steps are similar to the ones suggested by Seynnes and Cronin^14^. For further information, the reader is referred to this article. In the second analysis phase, a custom written function searches for the aponeuroses and measures the ACSA of the muscle. The function uses the defined outline-finder starting points and performs a scan along the line between the points. The scanning beams are orientated vertical, horizontal or circular, depending on the location of the outline-finder starting point and the selected muscle. If a pixel value is above the contrast threshold, the beam breaks and the coordinates of the pixel are saved. Then, the saved pixel coordinates are sorted clockwise to avoid overlap and then connected by generating a polygon (Fig. 1e). This step is optional and is only executed when ticked. The area of the polygon is measured representing the ACSA of the muscle (Fig. 1f).

### Data collection

The data used in this study was collected in 60 adolescent and adult high-level soccer players of both sexes [n = 46 males and 14 females, 17.8 years (14–25)]. One-hundred eighty ultrasound images (three per individual) were analysed. We used B-mode EFOV ultrasonography (ACUSON Juniper, SIEMENS Healthineers, Erlangen, Germany) with a 5.6 cm, linear-array probe (6.2–13.3 MHz, 12L3, Acuson 12L3) to assess the ACSA of RF and VL. We acquired pictures at rest while the participants laid in a supine position on their back. A guide was mounted to the leg in order to keep the same transversal path^19^. We took pictures of both muscles at 33 and 50% of the distance between the trochanter major and the lateral femur condyle. We acquired scans of both muscles either in the same picture or in two separate pictures, to test different modalities of the ACSAuto-script (see section “Program description”). Because of regional differences in muscle size and shape, 90 pictures from the proximal (33% of femur length) and 90 pictures from the mid (50% of femur length) region were included in the analysis. Therefore, 60 images were analysed per outline-finder starting point option. We measured ACSA of the RF and VL in all images using the ACSAuto script. We used the automatic scaling option during all measurements. Manual measurement of the ACSA of RF and VL was performed by an experienced investigator (investigator1) in FIJI^23^ and served as comparison. Manual measurement consisted of digitising the ACSA of each muscle using the polygon tool. To test inter and intra-investigator reliability, investigator1 and investigator2 evaluated a subsample of 30 pictures from the mid region for every modality and outline-finder starting point option. Furthermore, we conducted “Freerun” trials on all pictures where the suggested muscle outline was not manually corrected. We did this to test whether manual correction of the outline decreases the error of the program. To investigate whether manual and automatic scaling options of the ACSAuto program yield similar results, we compared a subsample of 30 manually scaled pictures from the mid region of the RF to the respective automatically scaled images. The study was approved by the regional ethics committee (Ethikkommission Zentral- und Nordwestschweiz; Project ID: 2017-02148) and complied with the Declaration of Helsinki. Participants signed an informed written consent prior to the start of the study after receiving all relevant study information. If participants were under 18, a parent or legal guardian signed the informed written consent prior to the study after receiving all relevant information.

### Statistics

All statistical analyses were performed in R software^28, 29^ (Base, BlandAltmanLeh and irr packages) and on an excel spreadsheet^28^. We compared ACSAuto measurements to manual measurements for all modalities and outline-finder starting point options. For this purpose, we calculated consecutive-pairwise intra-class correlations (ICC) and standard error of measurement (SEM) with 95% compatibility intervals (CI). Bland–Altman analysis^30^ was used to test the agreement between two analysis methods. Limits of agreement were set to ± 1.96 standard deviations (SD). The standardized mean bias was calculated according to Hopkins^28^, with 0.1, 0.3, 0.6, 1.0 and 2.0 being small, moderate, large, very large and extremely large errors. Additionally, we examined inter- and intra-investigator reliability for a subsample of 90 pictures from the mid region by calculating ICCs and SEMs with 95% CI. We calculated minimal detectable change (MDC) as $SEM\times 1.96\times \sqrt{2}$. We applied Bland–Altman analysis to test the agreement between analysis methods. We categorized standardized mean bias as 0.1, 0.3, 0.6, 1.0 and 2.0 for extremely high, very high, high, moderate low reliability^28^.

## Results

### Comparison of area measurement between ACSAuto and manual analysis

**Figure 2:**
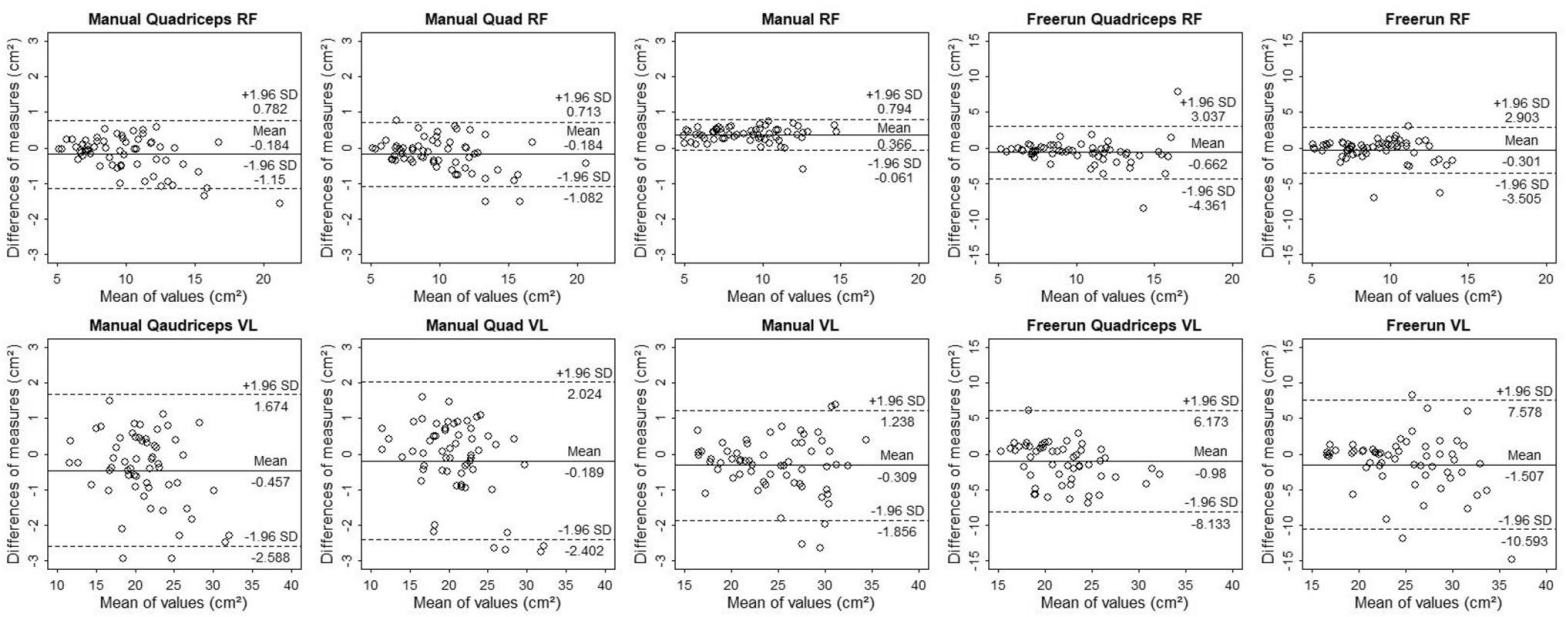
Bland–Altman plots of all modes using “Manual” outline-finder option compared to the manual measurement. M. rectus femoris (RF) and m. vastus lateralis (VL). The differences between measurements are plotted against measurement means. Dotted and solid lines illustrate 95% limits of agreement and bias. During the “Quadriceps” mode both muscles are analysed in one image. During the “Quad” mode, only one muscle is evaluated per image even though both muscles are displayed. During RF and VL modalities, both muscles were analysed in separate pictures. “Freerun” describes a trial where the suggested outlines were not manually corrected.

ICCs, SEMs, mean bias and standardized mean bias with 95% CI for all muscles and modes comparing manual to ACSAuto measurements are shown in Table 1 and Fig. 2. The analysis of “rectus femoris” and “vastus lateralis” across all outline-finder options showed the highest ICCs and lowest SEMs ranging from 0.982 to 0.998 and 0.10 to 0.74 cm^2^ respectively. Mean bias and standardized mean bias ranged from − 0.34 to 0.37 cm^2^ and − 0.12 to 0.17 SDs, resulting in small measurement errors. For the “quad RF” and “quad VL” modalities, ICCs were between 0.948 and 0.996 and SEMs between 0.29 and 0.96 cm^2^. Mean bias and standardized mean bias were between − 0.21 to − 0.08 cm^2^ and − 0.11 to 0.03 SDs, resulting in small measurement errors. ICCs for both muscles ranged from 0.947 to 0.996, SEMs from 0.27 to 0.94 cm^2^, mean bias from − 0.46 to 0.06 cm^2^ and standardized mean bias from -0.17 to 0.07 SDs, resulting in small errors for the “Quadriceps” modality. The “Freerun” modality resulted in ICCs from − 0.045 to 0.683 and SEMs from 1.84 to 5.23 cm^2^. Mean bias ranged from − 1.66 to 3.20 cm^2^ and standardized mean bias from − 0.66 to 1.57, demonstrating very large errors. Overall, RF measurements displayed higher ICCs and lower SEMs, mean bias and standardized mean bias compared to VL measurements (Table 1; Fig. 2). We found no obvious differences for ICC, SEM, mean bias and standardized mean bias in “Manual”, “Fixed Pixel” and “Automatic” options to define outline-finder starting points (Table 1). As shown in Table 1, we found negligible differences when comparing manual (ManRF) to automatic scaling options of the ACSAuto program. However, it seems that most analyses using the ACSAuto plugin yielded slightly smaller values compared to manual analysis (Table 1). Except for the “Quadriceps RF” modality using “Manual” outline-finder starting points, measured bias was not proportional to averaged values. This indicates that most manual and ACSAuto analyses agree equally throughout the measurement range (Fig. 2).

**Table 1:** Intra-class correlation (ICC), standard error of measurement (SEM) and standardized mean bias with 95% compatibility interval.

Modality	ICC	SEM	Mean bias	Standardized mean bias
**Quadriceps RF and VL**				
QaMRF	0.993 (0.989,0.996)	0.43 (0.36,0.52)	− 0.18 (− 1.15,0.78)	− 0.06 (− 0.10,− 0.02)
QaFRF	0.988 (0.981,0.993)	0.38 (0.32,0.46)	− 0.04 (− 1.11,1.04)	− 0.01 (− 0.06,0.03)
QaARF	0.983 (0.972, 0.99)	0.32 (0.27,0.39)	0.06 (− 1.10,1.21)	0.02 (− 0.03,0.07)
QaMVL	0.97 (0.951,0.982)	0.77 (0.65,0.94)	− 0.46 (− 2.59,1.67)	− 0.11 (− 0.17,− 0.04)
QaFVL	0.968 (0.947,0.981)	0.77 (0.66,0.94)	− 0.24 (− 2.46,1.97)	− 0.06 (− 0.12,0.01)
QaAVL	0.975 (0.958,0.985)	0.74 (0.63,0.91)	− 0.12 (2.10,1.85)	− 0.03 (− 0.09,0.03)
**Freerun quadriceps RF and VL**				
FREQaMRF	0.648 (0.469,0.775)	1.89 (1.60,2.32)	− 0.66 (− 4.36,3.04)	− 0.26 (− 0.46,− 0.07)
FREQaFRF	0.412 (0.174,0.604)	2.54 (2.15,3.12)	0.07 (− 4.67,5.17)	0.03 (− 0.29,0.35)
FREQaARF	0.411 (0.173,0.604)	2.54 (2.16,3.10)	0.31 (− 4.93,5.20)	0.15 (− 0.17,0.47)
FREQaMVL	0.415 (0.178,0.607)	3.55 (3.00,4.34)	− 0.81 (− 8.13,6.17)	− 0.33 (− 0.66,− 0.01)
FREQaFVL	0.269 (0.013,0.491)	3.69 (3.12,4.51)	3.20 (− 6.02,11.88)	1.17 (0.76,1.57)
FREQaAVL	0.351 (0.104,0.557)	3.88 (3.28,4.47)	1.59 (− 6.84,10.56)	0.56 (0.13,0.76)
**Quad RF and VL**				
QMRF	0.994 (0.99,0.996)	0.37 (0.31,0.45)	− 0.18 (− 1.08,0.71)	− 0.06 (− 0.10,− 0.02)
QFRF	0.989 (0.981,0.993)	0.36 (0.31,0.44)	− 0.11 (− 1.10,0.89)	− 0.03 (− 0.08,0.01)
QARF	0.981 (0.968, 0.988)	0.55 (0.47,0.67)	− 0.08 (− 1.43,1.27)	− 0.03 (− 0.08,0.03)
QMVL	0.969 (0.948,0.981)	0.79 (0.67,0.96)	− 0.19 (− 2.40,2.02)	− 0.04 (− 0.11,0.02)
QFVL	0.987 (0.978,0.992)	0.68 (0.58,0.83)	− 0.21 (− 2.10,1.68)	− 0.05 (− 0.11,0.01)
QAVL	0.987 (0.979,0.992)	0.69 (0.59,0.84)	− 0.18 (− 2.02,1.66)	− 0.04 (− 0.10,0.01)
**RF and VL**				
MRF	0.993 (0.988,0.996)	0.20 (0.17,0.24)	0.37 (− 0.06,0.79)	0.15 (0.12,0.17)
FRF	0.994 (0.99,0.996)	0.15 (0.13,0.19)	0.34 (− 0.02,0.71)	0.14 (0.12,0.16)
ARF	0.993 (0.988,0.996)	0.14 (0.12,0.17)	0.39 (0.03,0.74)	0.16 (0.14,0.17)
ManRF	0.998 (0.996,0.999)	0.12 (0.1,0.14)	0.17 (− 0.15,0.50)	0.07 (0.05,0.09)
MVL	0.992 (0.987,0.995)	0.66 (0.56,0.80)	− 0.31 (− 1.85,1.23)	− 0.07 (− 0.11,− 0.02)
FVL	0.989 (0.982,0.994)	0.60 (0.51,0.73)	− 0.34 (− 2.19,1.52)	− 0.07 (− 0.12,− 0.02)
AVL	0.993 (0.988,0.996)	0.60 (0.51,0.73)	− 0.28 (− 1.79,1.23)	− 0.06 (− 0.10,− 0.02)
**Freerun RF and VL**				
FREMRF	0.319 (0.072,0.528)	2.26 (1.92,2.76)	− 0.26 (− 3.51,2.90)	− 0.17 (− 0.57,0.23)
FREMVL	0.388 (0.15,0.583)	4.50 (3.82,5.49)	− 1.66 (− 10.59,7.58)	0.47 (0.11,0.83)

Mean bias with limits of agreement set to ± 1.96 standard deviations (SD). Values for SEM and mean bias are displayed in cm^2^ with standardized mean bias being displayed in SDs. All values calculated for m. rectus femoris (RF) and m. vastus lateralis (VL) comparing ACSAuto to manual measurements. “Quadriceps” (Qa), “Quad” (Q) and separate image modes were used. “Manual” (M), “Fixed pixels” (F) and “Automatic” (A) outline-finder starting points. Freerun (fre) and manual scaling (ManRF) trials were compared as well.

### Reliability of ACSAuto program

**Figure 3:**
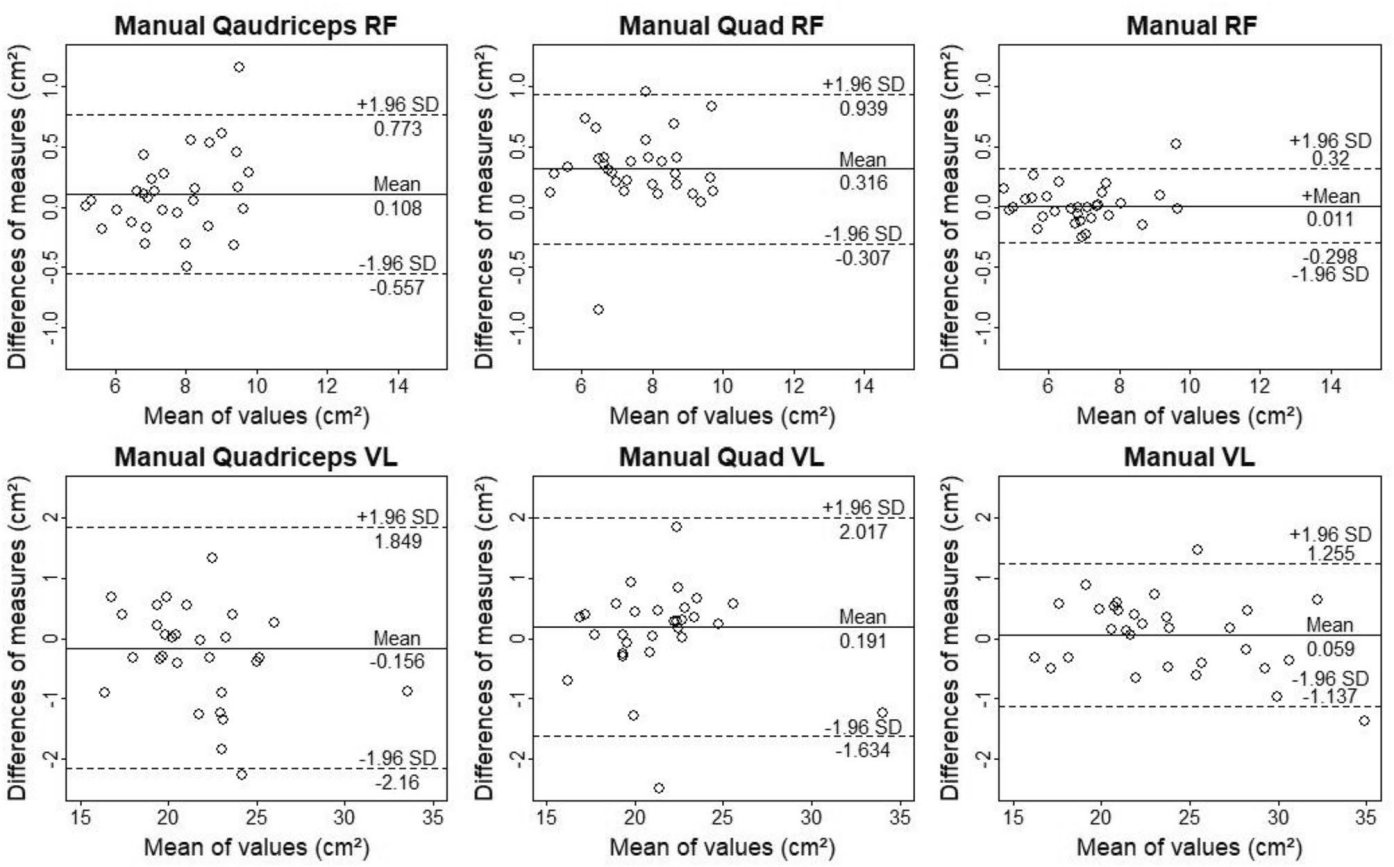
Bland–Altman plots of all modes using “Manual” outline-finder option comparing measurements of investigator1 to measurement of investigator2. M. rectus femoris (RF) and m. vastus lateralis (VL). The differences between measurements are plotted against measurement means. Dotted and solid lines illustrate 95% limits of agreement and bias. During the “Quadriceps” modalities both muscles are analysed in one image. During the “Quad” modalities, only one muscle is evaluated per image even though both muscles are displayed. During RF and VL modalities, both muscles were analysed in separate pictures.

**Figure 4:**
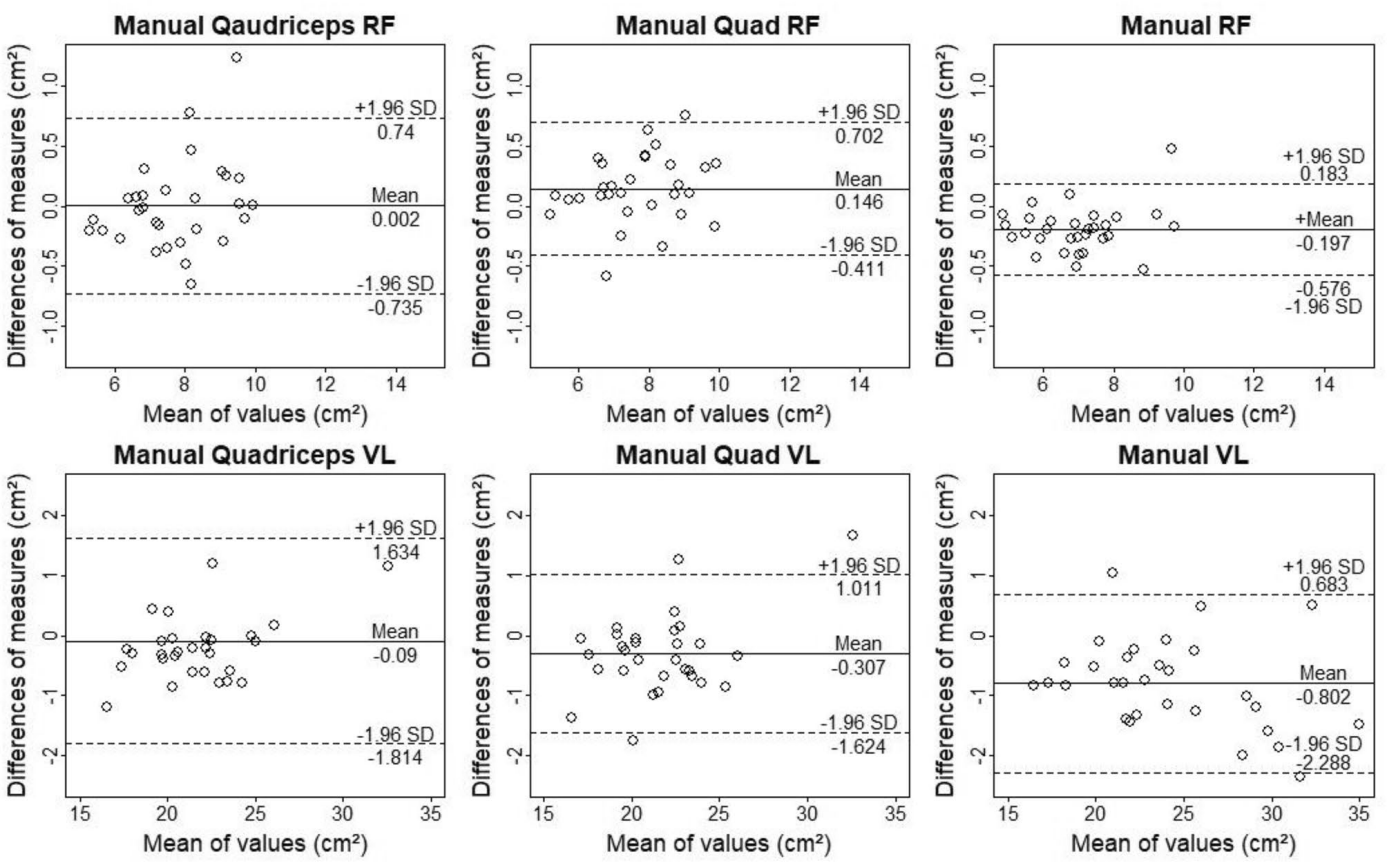
Bland–Altman plots of all modes using “Manual” outline-finder option comparing two measurements of investigator1. M. rectus femoris (RF) and m. vastus lateralis (VL). The differences between measurements are plotted against measurement means. Dotted and solid lines illustrate 95% limits of agreement and bias. During the “Quadriceps” modalities both muscles are analysed in one image. During the “Quad” modalities, only one muscle is evaluated per image even though both muscles are displayed. During RF and VL modalities, both muscles were analysed in separate pictures.

ICCs, SEMs, MDCs, mean bias and standardized mean bias with 95% CI for inter-rater comparisons are shown in Table 2 and Fig. 3. Inter-investigator comparison revealed ICCs, SEMs and MDCs ranging from 0.85 to 0.999, 0.07 to 1.16 cm^2^ and 0.22 to 2.4 cm^2^ respectively. Mean bias ranged from − 0.16 to 0.66 cm^2^ with standardized mean bias ranging from − 0.16 to 0.33, showing extremely high to high reliability. ICCs, SEMs, MDCs and standardized mean bias with 95% CI for intra-investigator comparisons are shown in Table 3. Results of Bland–Altman analysis can be seen in Fig. 4 and Table 3. Analyses revealed ICCs, SEMs and MDCs ranging from 0.883 to 0.999, 0.07 to 0.93 cm^2^ and 0.29 to 2.19 cm^2^ respectively. Mean bias ranged from − 0.80 to 0.15 cm^2^. Standardized mean bias ranged from − 0.26 to 0.19 showing extremely high to very high reliability. RF analysis of all modalities yielded higher ICCs and smaller SEMs, MDCs, mean bias and standardized mean bias compared to VL for both, inter and intra-investigator comparison (Tables 2, 3). We found no obvious differences between for ICC, SEM, MDCs mean bias and standardized mean bias between all outline-finder starting point options (see Tables 2, 3). Solely the “Quadriceps RF” modality using “Manual” outline-finder starting points raised concerns about homoscedasticity, because differences seem to be proportional to average value for inter and intra-investigator comparisons.

**Table 2:** Intra-class correlation (ICC) standard error of measurement (SEM) and standardized mean difference with 95% compatibility interval.

Variable	ICC	SEM	MDC	Mean bias	Standardized mean bias
**Quadriceps RF and VL**					
QaMRF	0.97 (0.938,0.986)	0.24 (0.19,0.32)	0.67	0.11 (− 0.56,0.77)	0.08 (− 0.01,0.18)
QaFRF	0.938 (0.873,0.97)	0.36 (0.29,0.48)	0.99	0.25 (− 0.75,1.24)	0.18 (0.04,0.33)
QaARF	0.934 (0.869,0.969)	0.35 (0.28,0.47)	1.04	0.11 (− 0.94,1.15)	0.04 (− 0.11,0.18)
QaMVL	0.958 (0.913,0.98)	0.72 (0.58,0.97)	2	− 0.16 (− 2.16,1.85)	− 0.05 (− 0.16,0.07)
QaFVL	0.926 (0.85, 0.964)	0.87 (0.69,1.16)	2.4	0.47 (− 1.93,− 2.87)	0.16 (0.00,0.31)
QaAVL	0.984 (0.967,0.993)	0.46 (0.37,0.62)	1.27	0.4 (− 0.87,1.67)	0.13 (0.05,0.21)
**Quad RF and VL**					
QMRF	0.972 (0.941,0.986)	0.22 (0.18,0.30)	0.62	0.32 (− 0.31,0.94)	0.24 (0.15,0.33)
QFRF	0.959 (0.915,0.98)	0.30 (0.24,0.40)	0.82	0.19 (− 0.63,1.00)	0.14 (0.02,0.25)
QARF	0.971 (0.940,0.986)	0.22 (0.18,0.30)	0.81	0.2 (− 0.61,1.01)	0.15 (0.03,0.27)
QMVL	0.958 (0.913,0.98)	0.66 (0.52,0.89)	1.82	0.19 (− 0.16,0.54)	0.06 (− 0.05,0.17)
QFVL	0.976 (0.949, 0.988)	0.49 (0.39,0.66)	1.36	0.66 (− 0.70,2.87)	0.22 (0.13,0.30)
QAVL	0.981 (0.96,0.991)	0.44 (0.35,0.59)	1.21	0.48 (− 0.74,1.69)	0.16 (0.08,0.23)
**RF and VL**					
MRF	0.996 (0.993,0.998)	0.11 (0.09,0.15)	0.31	0.01 (− 0.30,0.32)	0.01 (− 0.04,0.05)
FRF	0.998 (0.996,0.999)	0.09 (0.07, 0.13)	0.25	0.06 (− 0.19,0.30)	0.04 (0.00,0.08)
ARF	0.998 (0.996,0.999)	0.08 (0.07,0.11)	0.22	0.01 (− 0.21,0.24)	0.01 (− 0.02,0.04)
MVL	0.995 (0.991,0.998)	0.43 (0.35,0.58)	1.2	0.06 (− 1.14,1.26)	0.01 (− 0.04,0.06)
FVL	0.993 (0.985,0.997)	0.26 (0.21,0.35)	1.46	0.30 (− 1.16,1.76)	0.06 (0.03,0.09)
AVL	0.996 (0.992,0.998)	0.35 (0.28,0.48)	0.98	0.29 (− 0.69,1.27)	0.06 (0.02,0.10)

Mean bias with limits of agreement set to ± 1.96 standard deviations and minimal detectable change (MDC). Values are displayed in cm^2^ with standardized mean bias being displayed in SDs. All values calculated for m. rectus femoris (RF) and m. vastus lateralis (VL) comparing investigator1 and investigator2. “Manual” (M), “Fixed pixels” (F) and “Automatic” (A) outline-finder starting points. “Quadriceps” (Qa), “Quad” (Q) and separate image modes were used.

**Table 3:** Intra-class correlation (ICC) standard error of measurement (SEM) and standardized mean difference with 95% compatibility interval.

Modality	ICC	SEM	MDC	Mean bias	Standardized mean bias
**Quadriceps RF and VL**					
QaMRF	0.976 (0.951,0.988)	0.21 (0.17,0.29)	0.74	0.00 (− 0.74,0.74)	0.00 (0.21,0.36)
QaFRF	0.943 (0.883,0.972)	0.35 (0.28,0.47)	0.98	− 0.02 (− 0.95,1.00)	− 0.02 (− 0.15,0.12)
QaARF	0.951 (0.899,0.976)	0.30 (0.24,0.41)	0.91	− 0.15 (− 1.06,0.75)	− 0.12 (− 0.26,0.01)
QaMVL	0.990 (0.978, 0.995)	0.62 (0.26,0.93)	1.72	− 0.09 (− 1.81,1.63)	− 0.03 (− 0.13,0.08)
QaFVL	0.955 (0.908,0.978)	0.68 (0.54,0.91)	2.19	0.00 (− 2.19,2.19)	0.00 (− 0.14,0.14)
QaAVL	0.984 (0.967,0.993)	0.40 (0.32,0.54)	1.08	− 0.22 (− 1.30,0.86)	− 0.07 (0.14,0.01)
**Quad RF and VL**					
QMRF	0.980 (0.958,0.990)	0.19 (0.15,0.26)	0.56	0.15 (− 0.41,0.70)	0.11 (− 0.03,0.19)
QFRF	0.963 (0.923,0.982)	0.28 (0.22,0.37)	0.73	− 0.03 (− 0.76,0.70)	− 0.02 (− 0.13,0.08)
QARF	0.971 (0.941,0.986)	0.24 (0.19,0.32)	0.82	0.05 (− 0.86,0.77)	0.03 (− 0.08,0.15)
QMVL	0.984 (0.966,0.992)	0.48 (0.29,0.63)	1.32	− 0.31 (− 1.62,1.01)	− 0.10 (− 0.18,− 0.02)
QFVL	0.982 (0.962,0.991)	0.44 (0.35,0.60)	1.18	− 0.15 (− 1.33,1.04)	− 0.05 (− 0.12,0.03)
QAVL	0.985 (0.969,0.993)	0.39 (0.31,0.53)	1.07	− 0.13 (− 1.20,0.93)	− 0.04 (− 0.11,0.02)
**RF and VL**					
MRF	0.989 (0.978,0.995)	0.14 (0.11,0.19)	0.38	− 0.20 (− 0.58,0.18)	− 0.15 (− 0.21,− 0.10)
FRF	0.994 (0.987,0.997)	0.11 (0.08,0.14)	0.29	− 0.24 (− 0.53,0.05)	− 0.18 (− 0.22,− 0.14)
ARF	0.996 (0.991, 0.998)	0.09 (0.07,0.12)	0.24	− 0.22 (− 0.46,0.02)	− 0.16 (− 0.20,− 0.13)
MVL	0.986 (0.971,0.993)	0.57 (0.46,0.77)	1.49	− 0.80 (− 2.29,0.68)	− 0.17 (− 0.23,− 0.11)
FVL	0.992 (0.983,0.996)	0.44 (0.35,0.59)	1.56	− 0.74 (− 2.30,0.83)	− 0.16 (− 0.20,− 0.11)
AVL	0.993 (0.985,0.997)	0.41 (0.33,0.55)	1.13	− 0.51 (− 1.65,0.62)	− 0.11 (− 0.16,− 0.06)

Mean bias with limits of agreement set to ± 1.96 standard deviations and minimal detectable change (MDC). Values are displayed in cm^2^ with standardized mean bias being displayed in SDs. All values calculated for m. rectus femoris (RF) and m. vastus lateralis (VL) comparing two measurements of investigator 1. “Manual” (M), “Fixed pixels” (F) and “Automatic” (A) outline-finder starting points. “Quadriceps” (Qa), “Quad” (Q) and separate image modes were used.

## Discussion

We investigated the comparability and reliability of a novel semi-automatic tool to measure ACSA in EFOV ultrasound images of the RF and VL. Our results demonstrate very good agreement and small errors between manual and ACSAuto analysis, with mean bias and standardized mean bias smaller than 0.5 cm^2^ (4.3%) and 0.2 SDs respectively. Inter- and intra-investigator agreement was very good showing high reliability, with mean bias and standardized mean bias smaller than 1.0 cm^2^ (3.4%) and 0.4 SDs respectively. RF analyses yielded better results compared to VL analysis across all modalities and outline-finder options.

The “rectus femoris” and “vastus lateralis” modes were found to have the highest agreement and ICCs and lowest SEMs compared to manual analysis. Yet, standardized mean bias was slightly higher compared to the other modalities. Usually, image quality increases when images are acquired separately. As the length of the EFOV image in total is shorter, the muscle is displayed proportionally larger. Images with muscles displayed proportionally larger enable the user to better recognize the borders between muscle tissue and aponeurosis, increasing the accuracy of ACSA measurement. We implemented a zoom function in the script counteracting this issue. “Quadriceps”, “quad RF” and “quad VL” modalities showed slightly lower agreement and ICCs and higher SEMs when compared to manual measurement, whereas standardized mean bias were slightly lower than for “rectus femoris” and “vastus lateralis” modalities. Because sufficient contrast of muscle tissue and aponeurosis during single sweep images is more difficult to maintain, the outline-finding process might fail due to insufficient contrast and manual correction is needed. In a practical setting however, reduced amounts of images to acquire would be beneficial. Therefore, the contrast between muscle and aponeurosis tissue must be ample and aponeuroses clearly visible. This leads to improved detection of aponeuroses and outlines, thereby reducing amount, complexity and time of manual outline correction.

Comparing the “Freerun” to manual evaluation resulted in low agreement and large errors between measurements. We observed mean bias and standardized mean bias up to 3.2 cm^2^ and 1.17 SDs respectively. Low ICCs and high SEMs demonstrate the necessity of manual correction. This is important because outline-finding is dependent on image quality and therefore the expertise of the operator. Images of low quality will require more manual correction of the automatic outline-finding and thus increasing the subjective interpretation. As stated by Sennes and Cronin^14^, the detection of aponeuroses relies heavily on homogeneity of grey values and sufficient contrast. This might explain why RF measurements showed better agreement and reliability, as high image quality is easier to achieve due to the shape of the muscle.

The ACSAuto script seems to be reliable between and within investigators. Inter- and intra-investigator comparison revealed very good agreement and high to extremely high reliability for all modalities and muscles. Conversely, “rectus femoris” and “vastus lateralis” modalities showed highest mean bias and standardized mean bias, but lowest SEM. The “rectus femoris” modality resulted in lowest MDCs for RF between and within investigators. The “Quad VL” modality yielded lowest MDCs within investigators, whereas “vastus lateralis” modality resulted in lowest MDCs between investigators.

For comparison of ACSAuto measurement to manual as well as inter- and intra-investigator reliability measured bias was not proportional to averaged values, except for “Quadriceps RF” modality using “Manual” outline-finder starting points. This could be due to inferior image quality at the proximal scanning site for RF images. ACSA of the RF is larger at this site, leading to mean bias increases proportional to measurement means.

Recent randomized controlled trials reported RF ACSA adaptations between − 0.2 and 1.7 cm^2^ (− 2.9 and 18.5%) for six to fourteen weeks of training^18, 20, 21^. Reported VL ACSA increases ranged from 1.2 to 5.0 cm^2^ (7.4–17.1%) following six to ten weeks of training^18, 31, 21, 32^. Adaptations of RF are rather small and thus these adaptations might be hardly detectable because the MDC values for ACSAuto analyses were between 0.22 and 1.04 cm^2^. In contrast to that, adaptations of VL are large and likely good to evaluate with the ACSAuto plugin because MDC values were between 0.98 and 2.4 cm^2^. The inability to certainly detect small changes in the ACSA of a muscle following resistance training, is however unlikely due to errors in the ACSAuto script but potentially due to the variability of ultrasound measurements and manual evaluations in general^8, 18, 31^.

Although we found no differences between outline-finder starting point options, we advise users to apply the “Manual” option. As muscles are highly variable in their anatomical shape, options using pre-defined starting points might yield inferior outline detection. Comparing the “manual” and “automatic” scaling, we found high agreement and small errors between measurements, with mean bias equal to 0.17 cm^2^ and standardized mean bias smaller than 0.1 SDs. In this regard, automatic scaling can be used without compromising the accuracy of the measurement. Thereby, time effort and subjective influence by investigators can be reduced. When conducting ACSAuto analyses, time saving was higher for an experienced investigator than for an inexperienced investigator. The time saved was on average 3 min per 10 images using “Manual” outline-finder starting points for all modes except the “Quadriceps” mode. Image analysis using the “Quadriceps” mode took the same time as manual evaluation. In general, time saving was less for other outline-finder starting point options and is highly dependent on image quality.

In contrast to the fully automated TRAMA-algorithm developed by Salvi et al.^17^, our script allows for the evaluation of EFOV pictures. The TRAMA-algorithm^17^ is designed to measure the visible ACSA in static ultrasound images of several lower limb muscles. While this technique seems to be able to detect changes in muscle size and responses to musculoskeletal training^33, 34^, ACSA measurements in muscles exceeding the field of view of the ultrasound probe might be limited in meaningfulness. Chen et al.^15^ recently demonstrated an automatic ACSA segmenting algorithm using a deep learning model. Deep learning is a type of machine learning that uses a deep neural network^15^. The algorithm segments the ACSA of the RF in ultrasound images and test images were recorded during contraction of the muscle^15^. Deep learning rapidly turns out to be the state-of-the-art in medical image analysis^35^, and might be more powerful than the ACSAuto script proposed here. However, the algorithm of Chen et al.^15^ is only able to segment the ACSA of the RF and is limited in transferability to ultrasound images taken at rest. Other than dynamic ultrasound imaging during movements, most investigations record images in resting participants. In addition to that, none of the abovementioned articles supply information on how to implement the program for common use. Yet, this might be important to increase comparability among investigations assessing ACSA of lower limb muscles.

## Limitations

The following limitations of the ACSAuto script need to be mentioned. We compared the ACSAuto measurements to manual evaluation of ultrasound and not MRI images. The analysis is semiautomatic and therefore relies on subjective processing of the images. This limits objectivity and comparability among investigators^15–17^. In addition, we did not assess the between-day reliability and precision of our ultrasound measurements. So far, we only investigated EFOV ultrasound images of the RF and VL from highly trained individuals. Highly trained individuals have more muscle mass and less intramuscular fat than untrained persons, which might limit the reliability and comparability in other cohorts. Generally, ACSA measurements using the ACSAuto algorithm might be applicable for every muscle. Therefore the image quality (homogeneity of grey values and contrast^14^) must be high and outline-finding must be set to “Manual”. Some of the analysis parameters are hardcoded and most are based on our sample images. Not all these parameters can be adjusted without changing the script, limiting the robustness of the algorithm.

## Conclusion

In conclusion, we developed a reliable novel tool to assess the ACSA of RF and VL muscles that is comparable to manual analysis. Our results show, that ACSA measurement using the “rectus femoris” and “vastus lateralis” modalities yielded the best results. Additionally, the time effort needed for ACSA measurement can be reduced when using the ACSAuto script. Although semiautomatic, the ACSAuto script is free and openly accessible and can therefore partially reduce variability induced by manual analysis. In future investigations, more muscles need to be evaluated and the applicability of a deep learning model should be tested.

## Supplementary Information

Supplementary Information 1.

Supplementary Information 2.

Supplementary Information 3.

## Data Availability

The ACSAuto script, the dataset used for analysis, an installation guide and additional information for improved usage for the ACSAuto script are included in the supplementary information files. These materials are also available on github in the ACSAuto repository and can be accessed using the following link: https://github.com/PaulRitsche/ACSAuto.
