# Supplementary material for: ACSAuto-semi-automatic assessment of human vastus lateralis and rectus femoris cross-sectional area in ultrasound images
Source: Sci Rep. 2021 Jun 22;11:13042. doi: 10.1038/s41598-021-92387-6 (PMC8219722; doi:10.1038/s41598-021-92387-6)
Supplement: Supplementary file 1 — Supplementary Information 1. [file 41598_2021_92387_MOESM1_ESM.docx]

ACSAuto - semi-automatic assessment of human vastus lateralis and rectus femoris cross-sectional area in ultrasound images

**Paul Ritsche^1^*, Philipp Wirth^1^, Martino V. Franchi²^#2^, Oliver Faude^1#^**

^1^Department of Sport, Exercise and Health, University of Basel, 4052 Basel, Switzerland

²Institute of Physiology, Department of Biomedical Sciences, University of Padua, Padua, Italy

*Corresponding Author

Email: [Paul.ritsche@unibas.ch](mailto:Paul.ritsche@unibas.ch)

^#^ These authors share last authorship

///////////////////////////////////////////////////////////

//// ACSAuto - Anatomical Cross-Sectional Area Analysis ////

//////////////////////////////////////////////////////////

// ACSAuto - Analysis of anatomical cross-sectional area of muscles

// Copyright (C), 2020, Paul Ritsche & Philipp Wirth

// This Macro Requires:

// Canny Edge Detector plugin (https://imagej.nih.gov/ij/plugins/canny/index.html - Tom Gibara)

// Ridge Detection plugin v.1.4.0 (https://imagej.net/Ridge_Detection - Thorsten Wagner, Mark Hiner)

// This program is free software: you can redistribute it and/or modify

// it under the terms of the GNU General Public License as published by

// the Free Software Foundation, either version 3 of the License, or

// (at your option) any later version.

// This program is distributed in the hope that it will be useful,

// but WITHOUT ANY WARRANTY; without even the implied warranty of

// MERCHANTABILITY or FITNESS FOR A PARTICULAR PURPOSE. See the

// GNU General Public License for more details.

// You should have received a copy of the GNU General Public License

// along with this program. If not, see <http://www.gnu.org/licenses/>.

#@ String (value = "----- ACSAuto - Automatic cross-sectional area analysis -----", visibility="MESSAGE") title

#@ String (label = "Type of analysis", choices= {"Folder", "Image"}, persist=true, style="radioButtonHorizontal", description="Analyse single image or several images. Medial muscle border needs to be on the (top) left, lateral on the (bottom) right. If not, use flipping.") analysis

#@ Boolean (label = "Export to excel", value = FALSE, persist = TRUE, description = "Wheter to export results to excel file on desktop. If name not changed, values will be added.") excel

#@ String (value = "------------------ Folder analysis ------------------", visibility="MESSAGE") text1

#@ File (label = "Input directory", style = "directory") input

#@ File (label = "Output directory", style = "directory") output

#@ String (value = "--------------- Mode selection ---------------", visibility="MESSAGE") text3

#@ String (label = "Mode Name", choices = {"Rectus femoris", "Vastus lateralis", "Quad RF", "Quad VL", "Quadriceps"}, description="Select muscle(s) to be analysed on picture") muscle

#@ String (value = "--------------- Outline finder -------------", visibility="MESSAGE") text4

#@ String (label = " ", choices= {"Manual", "Automatic", "Fixed Pixels"}, style = "directory", persist=true, description="Select outline-finder strategy. Choose at Manual. Absolute fixed at Fixed Pixels. Relative fixed at Automatic") select_outline

#@ Boolean (label = "Sorting of coordinates", value = true, persist = false, describtion = "Whether to sort found outline coordinates to avoid overlap") sorting

#@ String (value = "---------------- Pixel scaling ----------------", visibility="MESSAGE") text5

#@ String (label ="Scale measurements", choices = {"Automatic", "Manual"}, style="radioButtonHorizontal", persist=true, description="Muscle scaling, produce output in cm²") scaling

#@ String (label="Scan depth (cm)", choices = {3, 3.5, 4, 4.5, 5, 5.5, 6, 6.5, 7, 7.5, 8, 8.5, 9}, persist=true, description="Requires images in input folder to be taken at same depth") depth

#@ Boolean (label ="Flip horizontally", value = false, persist = true, description = "Whether to flip the image horizontally") flip_horizontal

#@ Boolean (label ="Flip vertically", value = false, persist = true, description = "Whether to flip the image vertically") flip_vertical

// starting xys for outline finder

var starting_xy = newArray();

// length of the scale line

var lineLength = 0;

// sample by which the image is downsampled during preprocessing

var downSampleFac = 0.5;

// image ids

var IDraw;

var IDFoV;

var IDvessel1;

var IDvessel2;

var IDmask;

var IDScaling;

var IDScaling1;

var IDmaskScaling;

macro "ACSAutomated" {

//********************************

//********** FUNCTIONS ***********

//********************************

function getPreprocessingSettings(muscle) {

/*

Prompts user to adapt the preprocessing settings, default depends on muscle choice

*/

if (muscle == "Rectus femoris") {

defaultMinLengthFac = 0.6;

defaultTubenessSigma = 7;

defaultGaussianSigma= 0.5;

}

if (muscle == "Vastus lateralis") {

defaultMinLengthFac = 0.65;

defaultTubenessSigma = 7;

defaultGaussianSigma = 0.5;

}

if (muscle == "Quad RF") {

defaultMinLengthFac = 0.055;

defaultTubenessSigma = 3;

defaultGaussianSigma = 0;

}

if (muscle == "Quad VL") {

defaultMinLengthFac = 0.3;

defaultTubenessSigma = 7;

defaultGaussianSigma = 0;

}

if (muscle == "Quadriceps") {

defaultMinLengthFac = 0.055;

defaultTubenessSigma = 3;

defaultGaussianSigma = 0;

defaultMinLengthFac1 = 0.2;

defaultTubenessSigma1 = 7;

defaultGaussianSigma1 = 0;

}

if (muscle == "Quadriceps") {

Dialog.create("Preprocessing Settings");

Dialog.addNumber("Min Length Fac RF", defaultMinLengthFac);

Dialog.addNumber("Tubeness Sigma RF", defaultTubenessSigma);

Dialog.addNumber("Gaussian Sigma RF", defaultGaussianSigma);

Dialog.addNumber("Min Length Fac VL", defaultMinLengthFac1);

Dialog.addNumber("Tubeness Sigma VL", defaultTubenessSigma1);

Dialog.addNumber("Gaussian Sigma VL", defaultGaussianSigma1);

Dialog.show();

settings = newArray(6);

settings[0] = Dialog.getNumber();

settings[1] = Dialog.getNumber();

settings[2] = Dialog.getNumber();

settings[3] = Dialog.getNumber();

settings[4] = Dialog.getNumber();

settings[5] = Dialog.getNumber();

return settings;

}

else {

Dialog.create("Preprocessing Settings");

Dialog.addNumber("Min Length Fac", defaultMinLengthFac);

Dialog.addNumber("Tubeness Sigma", defaultTubenessSigma);

Dialog.addNumber("Gaussian Sigma", defaultGaussianSigma);

Dialog.show();

settings = newArray(3);

settings[0] = Dialog.getNumber();

settings[1] = Dialog.getNumber();

settings[2] = Dialog.getNumber();

return settings;

}

}

function flipImage(axis) {

/*

Flips image horizontally (axis == 0) or vertically (axis == 1)

*/

if (axis == 0) {

run("Flip Horizontally");

} else {

run("Flip Vertically");

}

}

function clearDisplay () {

/*

Clears display after getlinelength()

*/

roiManager("delete");

roiManager("show none");

close("Log");

close("ROI Manager");

close("Summary");

close("Junctions");

close("Threshold");

run("Close");

}

function excel_expo () {

/*

Export results to excel

*/

if (excel == true) {

run("Read and Write Excel", "stack_results no_count_column dataset_label=Analysis_results");

}

}

function getlineLength () {

/*

Scales image automatically

*/

// flip image

if (flip_horizontal) {

flipImage(0);

}

if (flip_vertical) {

flipImage(1);

}

if (scaling == "Automatic") {

if (muscle == "Quad RF" || muscle == "Quadriceps" || muscle == "Quad VL") {

run("Duplicate...", " ");

//pre-processing for ridge-detection

run("8-bit");

run("Threshold...");

setThreshold(50, 255);

setOption("BlackBackground", true);

run("Convert to Mask");

// select active image

IDScaling = getImageID();

selectImage(IDScaling);

n0 = nResults;

run("Analyze Particles...", "size=650-Infinity show=Masks");

n1 = nResults;

IJ.deleteRows(n0, n1);

// Subtract and close unnecessary pictures

IDmaskScaling = getImageID(); // Mask of shorter lines

imageCalculator("Subtract create", IDScaling, IDmaskScaling);

selectImage(IDmaskScaling); close();

selectImage(IDScaling); close();

// current image

IDScaling1 = getImageID();

run("Gaussian Blur...", "sigma=1.50");

n0 = nResults;

print(n0);

roiManager("show none");

run("Ridge Detection", "line_width=1 high_contrast=128 low_contrast=70 show_ids displayresults add_to_manager method_for_overlap_resolution=NONE sigma=0.79 lower_threshold=9 upper_threshold=18 minimum_line_length=175 maximum=280");

// length of scaling line in picture and number of pixels per cm

n1 = nResults;

print(n1);

Length = getResult("Length", n0+1);

print(Length);

lineLength = Length/depth;

IJ.deleteRows(n0, n1);

clearDisplay();

}

if (muscle == "Vastus lateralis") {

run("Duplicate...", " ");

// pre-processing for ridge-detection

run("8-bit");

run("Threshold...");

setThreshold(50, 255);

setOption("BlackBackground", true);

run("Convert to Mask");

IDScaling = getImageID();

selectImage(IDScaling);

n0 = nResults;

run("Analyze Particles...", "size=550-Infinity show=Masks Clear");

n1 = nResults;

IJ.deleteRows(n0, n1);

//Subtract and close unnecessary pictures

IDmaskScaling = getImageID(); // Mask of shorter lines

imageCalculator("Subtract create", IDScaling, IDmaskScaling);

selectImage(IDmaskScaling); close();

selectImage(IDScaling); close();

// current image

IDScaling1 = getImageID();

run("Gaussian Blur...", "sigma=1.50");

n0 = nResults;

print(n0);

run("Ridge Detection", "line_width=1 high_contrast=128 low_contrast=70 show_ids displayresults add_to_manager method_for_overlap_resolution=NONE sigma=0.79 lower_threshold=9 upper_threshold=18 minimum_line_length=200 maximum=500");

// Length of scaling line in picture and number of pixels per cm

n1 = nResults;

print(n1);

Length = getResult("Length", n0+1);

print(Length);

lineLength = Length/depth;

IJ.deleteRows(n0, n1);

clearDisplay();

}

if (muscle == "Rectus femoris") {

run("Duplicate...", " ");

// pre-processing for ridge detection

run("8-bit");

run("Threshold...");

setThreshold(50, 255);

setOption("BlackBackground", true);

run("Convert to Mask");

IDScaling = getImageID();

selectImage(IDScaling);

n0 = nResults;

run("Analyze Particles...", "size=1150-Infinity show=Masks");

n1 = nResults;

IJ.deleteRows(n0, n1);

//Subtract and close unnecessary pictures

IDmaskScaling = getImageID(); // Mask of shorter lines

imageCalculator("Subtract create", IDScaling, IDmaskScaling);

selectImage(IDmaskScaling); close();

selectImage(IDScaling); close();

// current image

IDScaling1 = getImageID();

run("Gaussian Blur...", "sigma=1.50");

n0 = nResults;

print(n0);

run("Ridge Detection", "line_width=1 high_contrast=128 low_contrast=70 show_ids displayresults add_to_manager method_for_overlap_resolution=NONE sigma=0.79 lower_threshold=9 upper_threshold=20 minimum_line_length=350 maximum=700");

// length of scaling line in picture and number of pixels per cm

n1 = nResults;

print(n1);

Length = getResult("Length", n0+1);

print(Length);

lineLength = Length/depth;

IJ.deleteRows(n0, n1);

clearDisplay();

}

}

}

function getFixedOutlineFinderStartingPoints(muscle) {

/*

Returns hardcoded starting points for the outline finder for each muscle

*/

if (muscle == "Rectus femoris") {

starting_xy = newArray(2);

starting_xy[0] = 170;

starting_xy[1] = 250;

}

if (muscle == "Vastus lateralis") {

starting_xy = newArray(6);

starting_xy[0] = 115;

starting_xy[1] = 180;

starting_xy[2] = 150;

starting_xy[3] = 340;

starting_xy[4] = 300;

starting_xy[5] = 400;

}

if (muscle == "Quad RF"){

starting_xy = newArray(2);

starting_xy[0] = 102;

starting_xy[1] = 240;

}

if (muscle == "Quad VL"){

starting_xy = newArray(6);

starting_xy[0] = 150;

starting_xy[1] = 340;

starting_xy[2] = 220;

starting_xy[3] = 360;

starting_xy[4] = 300;

starting_xy[5] = 335;

}

if (muscle == "Quadriceps"){

starting_xy = newArray(8);

starting_xy[0] = 102;

starting_xy[1] = 240;

starting_xy[2] = 150;

starting_xy[3] = 340; //325

starting_xy[4] = 220;

starting_xy[5] = 360;

starting_xy[6] = 310;

starting_xy[7] = 340;

}

}

function getAutoOutlineFinderStartingPoints(muscle) {

/*

Returns automatically estimated starting points for the outline finder for each muscle

*/

// determine region of interest

run("Duplicate...", " ");

IDcopy1 = getImageID();

run("8-bit");

run("Set Scale...", "distance=0 known=0 pixel=1 unit=pixel");

run("Convolve...", "text1=[-1 -1 -1 -1 -1\n-1 -1 -1 -1 -1\n-1 -1 24 -1 -1\n-1 -1 -1 -1 -1\n-1 -1 -1 -1 -1\n] normalize");

run("Median...", "radius=2");

run("Auto Local Threshold", "method=Median radius=15 parameter_1=0 parameter_2=0 white");

run("Options...", "iterations=2 count=1 black do=Close");

run("Analyze Particles...", "size=10000-Infinity add");

roiManager("Select", 0);

getSelectionBounds(x, y, width, height);

roiManager("delete");

selectImage(IDcopy1);

close();

// determine starting points

if (muscle == "Rectus femoris") {

starting_xy = newArray(2);

starting_xy[1] = round(x + 0.5*width);

starting_xy[0] = round(y + 0.35*height);

}

if (muscle == "Vastus lateralis") {

starting_xy = newArray(6);

starting_xy[0] = round(y + 0.2*height);

starting_xy[1] = round(x + 0.2*width);

starting_xy[2] = round(y + 0.35*height);

starting_xy[3] = round(x + 0.6*width);

starting_xy[4] = round(y + 0.7*height);

starting_xy[5] = round(x + 0.85*width);

}

if (muscle == "Quad RF") {

starting_xy = newArray(2);

starting_xy[0] = round(y + 0.15*height);

starting_xy[1] = round(x + 0.25*width);

}

if (muscle == "Quad VL") {

starting_xy = newArray(6);

starting_xy[0] = round(y + 0.3*height);

starting_xy[1] = round(x + 0.73*width);

starting_xy[2] = round(y + 0.5*height);

starting_xy[3] = round(x + 0.8*width);

starting_xy[4] = round(y + 0.75*height);

starting_xy[5] = round(x + 0.65*width);

}

if (muscle == "Quadriceps") {

starting_xy = newArray(8);

starting_xy[0] = round(y + 0.15*height);

starting_xy[1] = round(x + 0.25*width);

starting_xy[2] = round(y + 0.3*height);

starting_xy[3] = round(x + 0.73*width);

starting_xy[4] = round(y + 0.5*height);

starting_xy[5] = round(x + 0.8*width);

starting_xy[6] = round(y + 0.75*height);

starting_xy[7] = round(x + 0.65*width);

}

for (i = 0; i < starting_xy.length; i++) {

starting_xy[i] = downSampleFac * starting_xy[i];

}

}

function getManualOutlineFinderStartingPoints(muscle) {

/*

Lets the user choose the starting points for the outline finder

*/

if (muscle == "Rectus femoris") {

starting_xy = newArray(2);

setTool("point");

waitForUser("Select Outline Finder Starting Point.");

if (selectionType() > 0) {

Roi.getCoordinates(x, y);

do_continue = x.length != 1;

} else {

do_continue = 1;

}

while (do_continue) {

run("Select None");

setTool("point");

waitForUser("Select Outline Finder Starting Point.");

if (selectionType() > 0) {

Roi.getCoordinates(x, y);

do_continue = x.length != 1;

}

if (nImages > 0) {

do_continue = 1;

} else {

do_continue = 0;

}

}

Roi.getCoordinates(x, y);

}

if (muscle == "Vastus lateralis") {

starting_xy = newArray(6);

setTool("multipoint");

waitForUser("Select 3 Outline Finder Starting Points.");

if (selectionType() > 0) {

Roi.getCoordinates(x, y);

do_continue = x.length != 3;

} else {

do_continue = 1;

}

while (do_continue) {

run("Select None");

setTool("multipoint");

waitForUser("Select 3 Outline Finder Starting Points.");

if (selectionType() > 0) {

Roi.getCoordinates(x, y);

do_continue = x.length != 3;

}

if (nImages > 0) {

do_continue = 1;

} else {

do_continue = 0;

}

}

Roi.getCoordinates(x, y);

}

if (muscle == "Quad RF") {

starting_xy = newArray(2);

setTool("point");

waitForUser("Select Outline Finder Starting Point.");

if (selectionType() >= 0) {

Roi.getCoordinates(x, y);

do_continue = x.length != 1;

} else {

do_continue = 1;

}

while (do_continue) {

run("Select None");

setTool("point");

waitForUser("Select Outline Finder Starting Point.");

if (selectionType() >= 0) {

Roi.getCoordinates(x, y);

do_continue = x.length != 1;

}

if (nImages > 0) {

do_continue = 1;

} else {

do_continue = 0;

}

}

Roi.getCoordinates(x, y);

}

if (muscle == "Quad VL") {

starting_xy = newArray(6);

setTool("multipoint");

waitForUser("Select 3 Outline Finder Starting Points.");

if (selectionType() > 0) {

Roi.getCoordinates(x, y);

do_continue = x.length != 3;

} else {

do_continue = 1;

}

while (do_continue) {

run("Select None");

setTool("multipoint");

waitForUser("Select 3 Outline Finder Starting Points.");

if (selectionType() > 0) {

Roi.getCoordinates(x, y);

do_continue = x.length != 3;

}

if (nImages > 0) {

do_continue = 1;

} else {

do_continue = 0;

}

}

Roi.getCoordinates(x, y);

}

if (muscle == "Quadriceps"){

starting_xy = newArray(8);

setTool("multipoint");

waitForUser("Select 4 Outline Finder Starting Points.");

if (selectionType() > 0) {

Roi.getCoordinates(x, y);

do_continue = x.length != 4;

} else {

do_continue = 1;

}

while (do_continue) {

run("Select None");

setTool("multipoint");

waitForUser("Select 4 Outline Finder Starting Points.");

if (selectionType() > 0) {

Roi.getCoordinates(x, y);

do_continue = x.length != 4;

} if (nImages >= 1) {

do_continue = 1;

} else {

do_continue = 0;

}

}

Roi.getCoordinates(x, y);

}

for (i = 0; i < x.length; i++) {

starting_xy[2*i + 1] = x[i];

starting_xy[2*i] = y[i];

}

}

function sqDistance(x0, x1, y0, y1) {

/*

Return the squared distance between (x0, y0) and (x1, y1)

*/

return (x1 - x0) * (x1 - x0) + (y1 - y0) * (y1 - y0);

}

function swap(xs, ys, i, j) {

/*

Swaps the coordinates of xs and ys

*/

tmp_x = xs[i];

tmp_y = ys[i];

xs[i] = xs[j];

ys[i] = ys[j];

xs[j] = tmp_x;

ys[j] = tmp_y;

}

function sortCoordinatesClockwise(xs, ys) {

/*

Sorts depending on squared distance

*/

for (i = 0; i < xs.length - 1; i++) {

squared_distance_to_neighbor = sqDistance(xs[i], xs[i+1], ys[i], ys[i+1]);

for (j = i+1; j < xs.length - 1; j++) {

squared_distance = sqDistance(xs[i], xs[j] , ys[i], ys[j]);

if (squared_distance < squared_distance_to_neighbor) {

swap(xs, ys, i+1, j);

squared_distance_to_neighbor = squared_distance;

}

}

}

}

function measureRectusArea(nbeams, R, N, thresh) {

/*

Measures the area of the isolated rectus femoris

*/

xs = newArray(nbeams);

ys = newArray(nbeams);

// define x and y

x = starting_xy[1];

y = starting_xy[0];

// do a radial scan around (x,y)

count = circScanLine(x, y, xs, ys, 0, nbeams, R, N, thresh, 0, 360);

// select raw image and rescale outline

selectImage(IDFoV); close();

selectImage(IDraw);

// make selection and measure

upscaleAndLocalSearch(xs, ys, 4);

makeSelection("polygon", xs, ys);

run("Set Scale...", "distance=lineLength known=1 pixel=1 unit=cm");

waitForUser("Adjust Region of Interest. Click OK when done");

roiManager("add");

roiManager("measure");

roiManager("delete");

Overlay.addSelection("red", 4);

}

function measureVastusLatArea(nbeams, nsteps, R, N, thresh) {

/*

Measures the area of the isolated vastus lateralis

*/

xs = newArray(2 * (nbeams + nsteps + nsteps));

ys = newArray(2 * (nbeams + nsteps + nsteps));

// upper side

count = 0;

count = circScanLine(starting_xy[1], starting_xy[0], xs, ys, count, nbeams, R, 10000, thresh, 90, 270);

count = verticalScanLine(starting_xy[1], starting_xy[0], starting_xy[3], starting_xy[2], xs, ys, count, nsteps, R, N, thresh, 1);

count = horizontalScanLine(starting_xy[3], starting_xy[2], starting_xy[5], starting_xy[4], xs, ys, count, nsteps, R, N, thresh, 1);

count = circScanLine(starting_xy[5], starting_xy[4], xs, ys, count, nbeams, R, 10000, thresh, 0, 180);

count = horizontalScanLine(starting_xy[5], starting_xy[4], starting_xy[3], starting_xy[2], xs, ys, count, nsteps, R, N, thresh, -1);

count = verticalScanLine(starting_xy[3], starting_xy[2], starting_xy[1], starting_xy[0], xs, ys, count, nsteps, R, N, thresh, -1);

real_xs = newArray(count);

real_ys = newArray(count);

for (i = 0; i < count; i++) {

real_xs[i] = xs[i];

real_ys[i] = ys[i];

}

//switch to raw image

selectImage(IDFoV); close();

selectImage(IDraw);

// make selection and measure

upscaleAndLocalSearch(real_xs, real_ys, 32);

if (sorting == true){

sortCoordinatesClockwise(real_xs, real_ys);

}

makeSelection("polygon", real_xs, real_ys);

run("Set Scale...", "distance=lineLength known=1 pixel=1 unit=cm");

waitForUser("Adjust Region of Interest. Click OK when done");

roiManager("add");

roiManager("measure");

roiManager("delete");

Overlay.addSelection("red", 4);

}

function measureQuadRFArea(nbeams, R, N, thresh) {

/*

Measures the area of the rectus femoris

*/

xs = newArray(nbeams);

ys = newArray(nbeams);

// define x and y

x = starting_xy[1];

y = starting_xy[0];

count = circScanLine(x, y, xs, ys, 0, nbeams, R, N, thresh, 0, 360);

// switch to raw image

selectImage(IDFoV); close();

selectImage(IDraw);

// make selection and measure

upscaleAndLocalSearch(xs, ys, 32);

makeSelection("polygon", xs, ys);

run("Set Scale...", "distance=lineLength known=1 pixel=1 unit=cm");

run("To Selection");

run("Out [-]");

waitForUser("Adjust Region of Interest. Click OK when done");

roiManager("add");

roiManager("measure");

roiManager("delete");

Overlay.addSelection("red", 4);

}

function measureQuadVLArea(nbeams, nsteps, R, N, thresh) {

/*

Measures the area of the vastus lateralis

*/

xs = newArray(2 * (nbeams + nsteps + nsteps));

ys = newArray(2 * (nbeams + nsteps + nsteps));

// upper side

count = 0;

count = circScanLine(starting_xy[1], starting_xy[0], xs, ys, count, nbeams, R, 10000, thresh, 180, 360);

count = horizontalScanLine(starting_xy[1], starting_xy[0], starting_xy[3], starting_xy[2], xs, ys, count, nsteps, R, N, thresh, 1);

count = horizontalScanLine(starting_xy[3], starting_xy[2], starting_xy[5], starting_xy[4], xs, ys, count, nsteps, R, N, thresh, 1);

count = circScanLine(starting_xy[5], starting_xy[4], xs, ys, count, nbeams, R, 10000, thresh, 0, 180);

count = horizontalScanLine(starting_xy[5], starting_xy[4], starting_xy[3], starting_xy[2], xs, ys, count, nsteps, R, N, thresh, -1);

count = horizontalScanLine(starting_xy[3], starting_xy[2], starting_xy[1], starting_xy[0], xs, ys, count, nsteps, R, N, thresh, -1);

real_xs = newArray(count);

real_ys = newArray(count);

for (i = 0; i < count; i++) {

real_xs[i] = xs[i];

real_ys[i] = ys[i];

}

// switch to raw image

selectImage(IDFoV); close();

selectImage(IDraw);

//make selection and measure

upscaleAndLocalSearch(real_xs, real_ys, 32);

if (sorting == true){

sortCoordinatesClockwise(real_xs, real_ys);

}

makeSelection("polygon", real_xs, real_ys);

run("Set Scale...", "distance=lineLength known=1 pixel=1 unit=cm");

run("To Selection");

waitForUser("Adjust Region of Interest. Click OK when done");

roiManager("add");

roiManager("measure");

roiManager("delete");

Overlay.addSelection("red", 4);

}

function measureQuadricepsArea(nbeams_r, R_r, N_r, thresh_r, nbeams_v, nsteps_v, R_v, N_v, thresh_v) {

/*

Measures the area of both, rectus femoris and vastus lateralis

*/

// measure Rectus

rectus_xs = newArray(nbeams_r);

rectus_ys = newArray(nbeams_r);

// define x and y

x = starting_xy[1];

y = starting_xy[0];

count = circScanLine(x, y, rectus_xs, rectus_ys, 0, nbeams_r, R_r, N_r, thresh_r, 0, 360);

//measure Vastus

xs = newArray(2 * (nbeams_v + nsteps_v + nsteps_v));

ys = newArray(2 * (nbeams_v + nsteps_v + nsteps_v));

// upper side

count = 0;

count = circScanLine(starting_xy[3], starting_xy[2], xs, ys, count, nbeams_v, R_v, 10000, thresh_v, 180, 360);

count = horizontalScanLine(starting_xy[3], starting_xy[2], starting_xy[5], starting_xy[4], xs, ys, count, nsteps_v, R_v, N_v, thresh_v, 1);

count = horizontalScanLine(starting_xy[5], starting_xy[4], starting_xy[7], starting_xy[6], xs, ys, count, nsteps_v, R_v, N_v, thresh_v, 1);

count = circScanLine(starting_xy[7], starting_xy[6], xs, ys, count, nbeams_v, R_v, 10000, thresh_v, 0, 180);

count = horizontalScanLine(starting_xy[7], starting_xy[6], starting_xy[5], starting_xy[4], xs, ys, count, nsteps_v, R_v, N_v, thresh_v, -1);

count = horizontalScanLine(starting_xy[5], starting_xy[4], starting_xy[3], starting_xy[2], xs, ys, count, nsteps_v, R_v, N_v, thresh_v, -1);

real_xs = newArray(count);

real_ys = newArray(count);

for (i = 0; i < count; i++) {

real_xs[i] = xs[i];

real_ys[i] = ys[i];

}

// switch to raw image

selectImage(IDFoV); close();

selectImage(IDraw);

//measure rectus

upscaleAndLocalSearch(rectus_xs, rectus_ys, 32);

makeSelection("polygon", rectus_xs, rectus_ys);

run("Set Scale...", "distance=lineLength known=1 pixel=1 unit=cm");

run("To Selection");

run("Out [-]");

waitForUser("Adjust Region of Interest. Click OK when done");

roiManager("add");

roiManager("measure");

roiManager("delete");

Overlay.addSelection("red", 4);

//measure vastus

upscaleAndLocalSearch(real_xs, real_ys, 32);

if (sorting == true){

sortCoordinatesClockwise(real_xs, real_ys);

}

makeSelection("polygon", real_xs, real_ys);

run("Set Scale...", "distance=lineLength known=1 pixel=1 unit=cm");

run("To Selection");

waitForUser("Adjust Region of Interest. Click OK when done");

roiManager("add");

roiManager("measure");

roiManager("delete");

Overlay.addSelection("red", 4);

}

function circScanLine(x, y, xs, ys, count, nbeams, R, N, thresh, angle_start, angle_end) {

/*

Performs a scan along the radial axes around the point (x,y)

*/

// iterate over all beams

for (i = 0; i < nbeams; i++) {

// define the angle of the beam

perc_diff = (angle_end - angle_start) / 360;

perc_start = angle_start / 360;

perc_end = angle_end / 360;

theta = (perc_start + i * perc_diff / nbeams) * 2 * 3.14;

// increase radius stepwise

for (j = 0; j < N; j++) {

// current radius

r = R * j / N;

// pixels

xi = round(x + r*cos(theta));

yi = round(y + r*sin(theta));

xs[count] = x;

ys[count] = y;

// if pixel is above some threshold, add it to the list and break

if (getPixel(xi, yi) > thresh) {

xs[count] = xi;

ys[count] = yi;

j = N;

}

}

count ++;

}

return count;

}

function verticalScanLine(x0, y0, x1, y1, xs, ys, count, nsteps, R, N, thresh, sign) {

/*

Performs a scan along the line between (x0, y0) and (x1, y1) in the vertical direction

*/

for (i = 0; i < nsteps; i++) {

// x-position of the scan line

xi = round(i * (x1 - x0) / nsteps) + x0;

// scan y-positions

for (j = 0; j < N; j++) {

// calculate current y-position

r = R * j / N;

yi = round(i * (y1 - y0) / nsteps - sign * r) + y0;

xs[count] = xi;

ys[count] = round(i * (y1 - y0) / nsteps) + y0;

// if pixel is above some threshold, add it to the list and break

if (getPixel(xi, yi) > thresh) {

xs[count] = xi;

ys[count] = yi;

count++;

j = N;

}

}

}

return count;

}

function horizontalScanLine(x0, y0, x1, y1, xs, ys, count, nsteps, R, N, thresh, sign) {

/*

Performs a scan along the line between (x0, y0) and (x1, y1) in the horizontal direction

*/

for (i = 0; i < nsteps; i++) {

// x-position of the scan line

yi = round(i * (y1 - y0) / nsteps) + y0;

// scan y-positions

for (j = 0; j < N; j++) {

// calculate current y-position

r = R * j / N;

xi = round(i * (x1 - x0) / nsteps + sign * r) + x0;

ys[count] = yi;

xs[count] = round(i * (x1 - x0) / nsteps) + x0;

// if pixel is above some threshold, add it to the list and break

if (getPixel(xi, yi) > thresh) {

xs[count] = xi;

ys[count] = yi;

count++;

j = N;

}

}

}

return count;

}

function upscaleAndLocalSearch(xs, ys, window_size) {

/*

Rescales the points in xs and ys to fit the original image. Replace coordinates by coordinates of near-by maximum intensity.

*/

for (i = 0; i < xs.length; i++) {

new_x = round(xs[i] / downSampleFac);

new_y = round(ys[i] / downSampleFac);

current_max = getPixel(new_x, new_y);

left = new_x - window_size / 2;

right = new_x + window_size / 2;

top = new_y - window_size / 2;

bottom = new_y + window_size / 2;

for (xi = left; xi < right; xi ++) {

for (yi = top; yi < bottom; yi ++) {

if (getValue(xi, yi) > current_max) {

new_x = xi;

new_y = yi;

current_max = getValue(xi, yi);

}

}

}

xs[i] = new_x;

ys[i] = new_y;

}

}

function processImages(input_dir, settings) {

/*

Processes a batch of images

*/

// get list of input files and iterate through them

file_list = getFileList(input_dir);

for (i=0; i < file_list.length; i++) {

open(file_list[i]);

if (scaling == "Automatic") {

getlineLength();

}

if (scaling == "Manual") {

run("Select None");

setTool("line");

waitForUser("Select scaling line. Click OK when done");

getLine(x1, y1, x2, y2, lineWidth);

Length = sqrt(pow(x1-x2, 2) + pow(y1-y2, 2));

lineLength = Length/depth;

}

// set the outline finder starting points automatically

if (select_outline == "Automatic") {

getAutoOutlineFinderStartingPoints(muscle);

}

// process a single image

if (muscle == "Quadriceps") {

run("Duplicate...", " ");

getAutoOutlineFinderStartingPoints("Quad RF");

preProcessImage(settings[0], settings[1], settings[2]);

processImage("Quad RF");

close();

getAutoOutlineFinderStartingPoints("Quad VL");

preProcessImage(settings[3], settings[4], settings[5]);

processImage("Quad VL");

} else{

if (select_outline == "Automatic") {

getAutoOutlineFinderStartingPoints(muscle);

}

preProcessImage(settings[0], settings[1], settings[2]);

processImage(muscle);

}

// save image with overlay and close it

run("Flatten");

save(output + File.separator + File.name);

close();

}

}

function preProcessImage(minLengthFac, tubenessSigma, gaussianSigma) {

/*

Preprocesses a single image

*/

// get image id, width and height

IDraw = getImageID();

W = getWidth;

H = getHeight;

// filter and detect fascia

run("Duplicate...", " ");

IDFoV = getImageID();

// downsample

newWidth = round(downSampleFac * W);

newHeight = round(downSampleFac * H);

run("Size...", "Width = newWidth Height = newHeight Depth = 1");

WFoV = getWidth; //Dimensions of the field of view

HFoV = getHeight;

minLength = minLengthFac * WFoV;

// preprocessing

run("8-bit");

run("Set Scale...", "distance=0 known=0 pixel=1 unit=pixel");

run("Subtract Background...", "rolling=50 sliding");

run("Non-local Means Denoising", "sigma=15 smoothing_factor=1 auto");

run("Bandpass Filter...", "filter_large=40 filter_small=3 suppress=None tolerance=5 saturate");

run("Enhance Local Contrast (CLAHE)", "blocksize=36 histogram=256 maximum=4 mask=*None* fast_(less_accurate)");

run("Tubeness", "sigma=tubenessSigma");

if (gaussianSigma > 0) {

run("Gaussian Blur...", "Sigma(Radius)=gaussianSigma");

}

IDvessel1 = getImageID();

selectImage(IDvessel1);

run("8-bit");

run("Canny Edge Detector", "gaussian=2 low=3 high=9.75");

run("Analyze Particles...", "size=0-minLength show=Masks");

IDmask = getImageID(); // Mask of shorter lines

imageCalculator("Subtract create", IDvessel1, IDmask);

// close unnecessary images

selectImage(IDmask); close();

selectImage(IDvessel1); close();

// current image

IDvessel2 = getImageID();

}

function processImage(muscle) {

/*

Processes a single image

*/

// this asks for elliptical selections which are set to a pixel value of 0

// (let's user delete artifacts)

setTool("ellipctical");

setColor(0);

waitForUser("Select area. Click OK to delete.");

while(selectionType() >= 0) { // 0 for rectangle

Roi.getContainedPoints(xpoints, ypoints);

for (i = 0; i < xpoints.length; i++) {

setPixel(xpoints[i], ypoints[i], 0);

}

run("Select None");

waitForUser("Select area. Click OK to delete.");

}

// this asks for a point selection which is used to find outlines

if (select_outline == "Fixed Pixels") {

getFixedOutlineFinderStartingPoints(muscle);

} else if (select_outline == "Manual") {

getManualOutlineFinderStartingPoints(muscle);

} else {

// Automatic

}

// depending on muscle type, area is evaluated differently

if (muscle == "Rectus femoris") {

measureRectusArea(40, 200, 10000, 0);

} else if (muscle == "Vastus lateralis") {

measureVastusLatArea(10, 10, 75, 50, 0);

} else if (muscle == "Quad RF") {

measureQuadRFArea(30, 200, 10000, 0);

} else if (muscle == "Quad VL") {

measureQuadVLArea(11, 11, 75, 1000, 0);

} else if (muscle == "Quadriceps"){

measureQuadricepsArea(30, 200, 10000, 0, 11, 11, 75, 1000, 0);

}

// close IDvessel2 and select current image

selectImage(IDvessel2); close();

selectImage(IDraw);

// save image with overlay and close it

run("Flatten");

save(output + File.separator + File.name);

close();

}

//********************************

//************ MAIN **************

//********************************

// settings

settings = getPreprocessingSettings(muscle);

// process single image

if (analysis == "Image") {

if (scaling == "Automatic") {

getlineLength();

}

if (scaling == "Manual") {

if (flip_horizontal) {

flipImage(0);

}

if (flip_vertical) {

flipImage(1);

}

run("Select None");

setTool("line");

waitForUser("Select scaling line. Click OK when done");

getLine(x1, y1, x2, y2, lineWidth);

Length = sqrt(pow(x1-x2, 2) + pow(y1-y2, 2));

lineLength = Length/depth;

}

if (select_outline == "Automatic") {

getAutoOutlineFinderStartingPoints(muscle);

}

if (muscle == "Quadriceps") {

run("Duplicate...", " ");

getAutoOutlineFinderStartingPoints("Quad RF");

preProcessImage(settings[0], settings[1], settings[2]);

processImage("Quad RF");

close();

getAutoOutlineFinderStartingPoints("Quad VL");

preProcessImage(settings[3], settings[4], settings[5]);

processImage("Quad VL");

}

else {

preProcessImage(settings[0], settings[1], settings[2]);

processImage(muscle);

}

excel_expo();

run("Close All");

}

//process all files in the input directory

else {

processImages(input, settings);

}

excel_expo();

close("ROI Manager");

run("Close All");

}
