# Supplementary material for: ACSAuto-semi-automatic assessment of human vastus lateralis and rectus femoris cross-sectional area in ultrasound images
Source: Sci Rep. 2021 Jun 22;11:13042. doi: 10.1038/s41598-021-92387-6 (PMC8219722; doi:10.1038/s41598-021-92387-6)
Supplement: Supplementary file 2 — Supplementary Information 2. [file 41598_2021_92387_MOESM2_ESM.docx]

ACSAuto - semi-automatic assessment of human vastus lateralis and rectus femoris cross-sectional area in ultrasound images

**Paul Ritsche^1^*, Philipp Wirth^1^, Martino V. Franchi² ^#2^, Oliver Faude^1 #^**

^1^Department of Sport, Exercise and Health, University of Basel, Basel, Switzerland

²Institute of Physiology, Department of Biomedical Sciences, University of Padua, Padua, Italy

*Corresponding Author

Email: [Paul.ritsche@unibas.ch](mailto:Paul.ritsche@unibas.ch)

^#^ These authors share last authorship

# Installation guide

In order to use the ACSAuto script, it needs to be installed as a plugin in FIJI. If you are not familiar with FIJI and/or need to install it, please take a look at this link: https://fiji.sc/

There are two ways how to install ACSAuto as a plugin in FIJI:

1. Clone the git repository:

git clone https://github.com/PaulRitsche/ACSAuto

Once you have downloaded the ACSAuto.ijm file on your local server, you need to open FIJI. Then you need to install the ACSAuto script as a plugin. Choose 'Plugins' -> 'Install' -> select the ACSAuto.ijm and Canny_Edge_Detector.class files -> 'open'.

Restart FIJI and the ACSAuto plugin will appear at the bottom of the FIJI plugin list and is ready to use.

2. Use ImageJ update sites (updates will be automatically installed):

Subsequently to downloading FIJI, you need to add the update site of the ACSAuto plugin as well as the sites of the dependencies.

Open FIJI and choose 'Help' -> 'Update' -> 'Manage update sites'. Now tick the boxes of the 'BIG-EPFL', 'Biomedgroup' and 'ResultsToExcel' update sites.

In doing so, dependencies and update releases will be automatically installed. Scroll to the bottom of the list and click 'Add update site'. A new update site should have been added. Modify the details of the new sites with double click:

Name: ACSAuto

URL: http://sites.imagej.net/ACSAuto/

Host: webdav:PRitsche

After entering the details, ensure ticking the box left of ACSAuto. Choose 'Close' -> 'Apply changes'. Now you have to re-start FIJI in order for the newly added plugins to be installed.

The ACSAuto plugin will appear at the bottom of the FIJI plugin list and is ready to use.
