# Supplementary material for: ACSAuto-semi-automatic assessment of human vastus lateralis and rectus femoris cross-sectional area in ultrasound images
Source: Sci Rep. 2021 Jun 22;11:13042. doi: 10.1038/s41598-021-92387-6 (PMC8219722; doi:10.1038/s41598-021-92387-6)
Supplement: Supplementary file 3 — Supplementary Information 3. [file 41598_2021_92387_MOESM3_ESM.docx]

ACSAuto - semi-automatic assessment of human vastus lateralis and rectus femoris cross-sectional area in ultrasound images

**Paul Ritsche^1^*, Philipp Wirth^1^, Martino V. Franchi² ^#2^, Oliver Faude^1 #^**

^1^Department of Sport, Exercise and Health, University of Basel, Basel, Switzerland

²Institute of Physiology, Department of Biomedical Sciences, University of Padua, Padua, Italy

*Corresponding Author

Email: [Paul.ritsche@unibas.ch](mailto:Paul.ritsche@unibas.ch)

^#^ These authors share last authorship

# Additional information for improved usage

In the following, additional information on how to use the ACSAuto plugin as efficient as possible can be found. The information is structured based on the workflow of the script. For general information on how to use the ACSAuto plugin please take a look at the supplementary instructional video.

The evaluation of ultrasound images by ACSAuto plugin is highly dependent on sufficient contrast between different tissues and homogeneity of grey values. When acquiring ultrasound images, aponeuroses should be clearly distinguishable from muscle tissue, especially at the medial and lateral muscle end.

Before analyzing ultrasound images, consider adding a *Shortcut* for the ACSAuto plugin. Click *Plugins* in the main menu, choose ‘Shortcuts’ -> ‘Add Shortcut’*.* Now you have to select the ACSAuto plugin and choose a shortcut on the keyboard. This step allows to easily use the ACSAuto plugin via the selected shortcut without choosing it from the plugin pull-down list.

Before you start analyzing your images, test the pre-specified pre-processing parameters. Adapt them to the characteristics of your images. If different, enter them every time you run the script. If batch mode is selected, the pre-processing settings will be applied to all images inside the selected folder.

Single images can be evaluated by dragging them into the FIJI main menu window and subsequently running the script on the active image.

Outline-finder starting points using the “Manual” modality should be placed in the middle of the selected outlines as well as near the medial and lateral end (if more than one point must be specified). When analyzing the m. vastus lateralis and the sorting clockwise option is ticked, place the outline-finder starting point in the muscle middle near the superficial aponeurosis. If starting points are placed with too little space between them, suggested outlines might be incorrect and overlap.

It is possible that, while adjusting the suggested outlines or ROI, the selection vanishes due to a misplaced click. CTRL+SHIFT+E can be used to get the last active selection back on the active image. In case the suggested outlines are inacceptable, a ROI can be manually drawn into the active image. Therefore, the *polygon tool* must be selected from the *FIJI* main menu. However, this should be avoided because it would increase the subjective influence of the investigator.
